# Clinical, radiographic, and histological evaluation of autogenous demineralized dentin matrix versus xenograft in alveolar ridge preservation: a randomized controlled trial

**DOI:** 10.1186/s40729-026-00682-6

**Published:** 2026-05-22

**Authors:** Yin Xu, Hui Zhang

**Affiliations:** 1 Department of Stomatology, People’s Hospital of China Medical University (Liaoning Provincial People’s Hospital), No. 33 Wenyi Road, Shenhe District Shenyang, 110016 China; 2 School of Stomatology, China Medical University, No. 77 Puhe Road, Shenbei New District Shenyang, 110122 China

**Keywords:** Alveolar ridge preservation, Demineralized dentin matrix, Autogenous tooth graft, Bone remodeling, Cone-beam computed tomography, Randomized controlled trial

## Abstract

**Purpose:**

This randomized controlled trial aimed to evaluate the clinical and histological efficacy of autogenous demineralized dentin matrix (DDM) compared with deproteinized bovine bone mineral (DBBM) for alveolar ridge preservation (ARP), with a specific focus on dimensional stability and remodeling dynamics over a 6-month follow-up period.

**Methods:**

Fifty patients requiring single-tooth extraction were randomized to receive ARP with either DDM (test group, n = 25) or DBBM (control group, n = 25). Dimensional changes were assessed by CBCT at baseline, 3 months, and 6 months. Histomorphometric analysis was performed on bone core biopsies harvested at 6 months.

**Results:**

Fifty patients were enrolled and randomly assigned to the test (DDM, n = 25) or control (DBBM, n = 25) group. At 3 months post-surgery, the test group exhibited significantly greater horizontal bone resorption at the mid-socket level (50%) compared with the control group (*p* = 0.006). However, at 6 months, no statistically significant differences were observed between the two groups regarding horizontal width reduction at any level (coronal, middle, apical) or vertical height reduction (*p* > 0.05). Histomorphometric analysis at 6 months revealed active new bone formation and good integration of graft particles in the DDM group, confirming its biodegradability and osteoconductive potential.

**Conclusions:**

Although autogenous DDM exhibited a faster remodeling rate and dimensional contraction in the early healing phase (3 months), it achieved long-term (6-month) dimensional stability comparable with that of the gold-standard xenograft. Furthermore, histological evidence of superior tissue integration suggests that DDM is a biologically viable and cost-effective alternative for alveolar ridge preservation.

## Background

According to the World Health Organization (WHO), oral diseases affect approximately 3.5 billion people worldwide [1], with caries and periodontal disease being the primary causes of tooth loss. Issues with masticatory function, phonetics, and esthetics also exert a significant negative impact on the patient´s psychological well-being and quality of life [2]. With the advancement of dental technology, implant-supported prostheses have become the preferred treatment modality for edentulism, with an estimated 20 million implants placed globally each year. However, the long-term success of implant therapy is fundamentally dependent on the availability of adequate bone volume and quality at the recipient site.

The alveolar process is a tooth-dependent tissue; its development and maintenance rely on physiological stimulation from the teeth. Following tooth extraction, the disruption of blood supply to the bundle bone inevitably leads to irreversible physiological resorption and dimensional alterations of the alveolar ridge [3]. Schropp et al. [4] reported that rapid remodeling occurs within the first 3 months post-extraction and continues up to 6 months. According to landmark systematic reviews, post-extraction healing is characterized by pronounced osseous resorption. The horizontal width reduction has been reported to range from 3.79 to 3.87 mm, while the vertical height loss typically exceeds 1 mm within the first 6 months [5, 6]. This substantial volumetric loss poses significant challenges for subsequent implant placement. Consequently, alveolar ridge preservation (ARP) at the time of extraction has become a standard clinical protocol for minimizing ridge atrophy. Currently, deproteinized bovine bone mineral (DBBM) is widely considered the gold standard xenograft for ARP due to its volume stability [7]. However, DBBM is primarily osteoconductive and exhibits a slow biodegradation rate, which may interfere with the quality of bone-implant contact (BIC).

In search of an ideal graft material that combines volume stability with regenerative potential, autogenous demineralized dentin matrix (DDM) has emerged as a promising alternative. Embryologically, teeth and alveolar bone share a common origin from neural crest cells, resulting in a high degree of biochemical similarity [8, 9]. Studies have confirmed that the inorganic component of dentin is primarily low-crystalline hydroxyapatite (HA) and tricalcium phosphate (TCP), which facilitates resorption by osteoclasts and participation in the remodeling process.

Ideally, the grafting material should possess osteoinductive properties. DDM retains a rich reservoir of bioactive molecules preserved within the mineralized matrix. Studies have shown that the dentin matrix contains type I collagen and various non-collagenous proteins, particularly bone morphogenetic proteins (BMPs), transforming growth factor-β (TGF-β), and osteopontin (OPN) [10]. Upon demineralization, these growth factors are exposed and released, recruiting mesenchymal stem cells and inducing osteogenic differentiation. Structurally, the inherent dentinal tubules (typically 2–4 μm in diameter) create a natural microporous scaffold [11]. This interconnected network facilitates angiogenesis and nutrient transport, further supporting the bone regeneration process.

Although pre-clinical and case studies have demonstrated the osteogenic capacity of DDM, high-level evidence directly comparing its clinical and histological outcomes with the gold standard (DBBM) in alveolar ridge preservation remains limited. Therefore, this randomized controlled trial (RCT) aimed to systematically evaluate the dimensional stability, bone remodeling dynamics, and histological quality of newly formed bone using autogenous DDM versus DBBM, providing scientific evidence for the clinical recycling of extracted teeth.

## Methods

### Study design and ethics

This study was designed as a prospective, randomized controlled clinical trial (RCT) to evaluate the efficacy of autogenous demineralized dentin matrix (DDM) for alveolar ridge preservation (ARP). The protocol adhered to the principles of evidence-based medicine and the Declaration of Helsinki (2013 revision). Ethical approval was granted by the Ethics Committee of The People’s Hospital of Liaoning Province (Approval No. 2025-H006). Written informed consent was obtained from all participants after a detailed explanation of the treatment plan and study objectives.

### Participants and grouping

A total of 50 patients requiring single-tooth extraction were recruited from the Department of Stomatology, The People’s Hospital of Liaoning Province, between February 2025 and June 2025. The cohort included 21 males and 29 females, with an age range of 21 to 70 years (mean age: 47.2 years).

### Randomization, allocation concealment, and blinding

Randomization was performed using a computer-generated random number sequence. The group allocation for each participant was concealed in sequentially numbered, opaque, sealed envelopes (SNOSE) prepared by an independent research assistant who was not involved in the surgical procedures. The envelopes were opened only after the atraumatic tooth extraction was completed and the socket integrity was verified. This study utilized a single-blind design. While the surgeon could not be blinded due to the distinct physical appearance of the graft materials, the outcome assessors (radiographic and histological examiners) were strictly blinded to the group allocation.

Patients were randomly assigned to two groups:

- *Control group (n* = *25):* The extraction sockets were grafted with deproteinized bovine bone mineral (DBBM; Bio-Oss®, Geistlich Pharma AG, Wolhusen, Switzerland).
- *Test group (n* = *25):* The sockets were grafted with autogenous DDM derived from the extracted teeth.

### Inclusion and exclusion criteria

#### Inclusion criteria

1. Age between 18 and 70 years, good general health;
2. Presence of a single non-restorable premolar or molar (tooth positions 4–7) with adjacent teeth present;
3. Presence of at least 3 mm of residual bone height apically to ensure primary stability;
4. Availability of sufficient autogenous tooth structure for DDM preparation;
5. Absence of acute infection or suppuration at the extraction site;
6. Willingness to comply with the follow-up protocol.

#### Exclusion criteria

1. Uncontrolled systemic diseases (e.g., hypertension, diabetes);
2. Heavy smoking (> 10 cigarettes/day);
3. Long-term use of medications affecting bone metabolism (e.g., bisphosphonates, corticosteroids) within the past 6 months;
4. History of head and neck radiation therapy;
5. Metabolic bone diseases or local bone pathologies (e.g., cysts, tumors);
6. Severe untreated periodontal disease or poor oral hygiene;
7. Pregnancy or lactation;
8. Participation in other clinical trials within the last 3 months.

### Sample size calculation

The sample size was calculated based on the estimates of variability and effect size derived from previous studies investigating dimensional changes after alveolar ridge preservation [12, 13]. To detect a clinically significant difference in ridge width reduction between groups (power = 0.80, α = 0.05), a minimum of 20 subjects per group was required. Considering a potential dropout rate of 20%, a total of 50 patients (25 per group) were enrolled in this study.

### Surgical procedure

All procedures were performed by the same experienced surgeon. Following local anesthesia, minimally invasive tooth extraction was performed without flap elevation to preserve the buccal and lingual bone plates. Thorough curettage was performed to remove granulation tissue.

#### Test group (DDM)

**Fig. 1:**
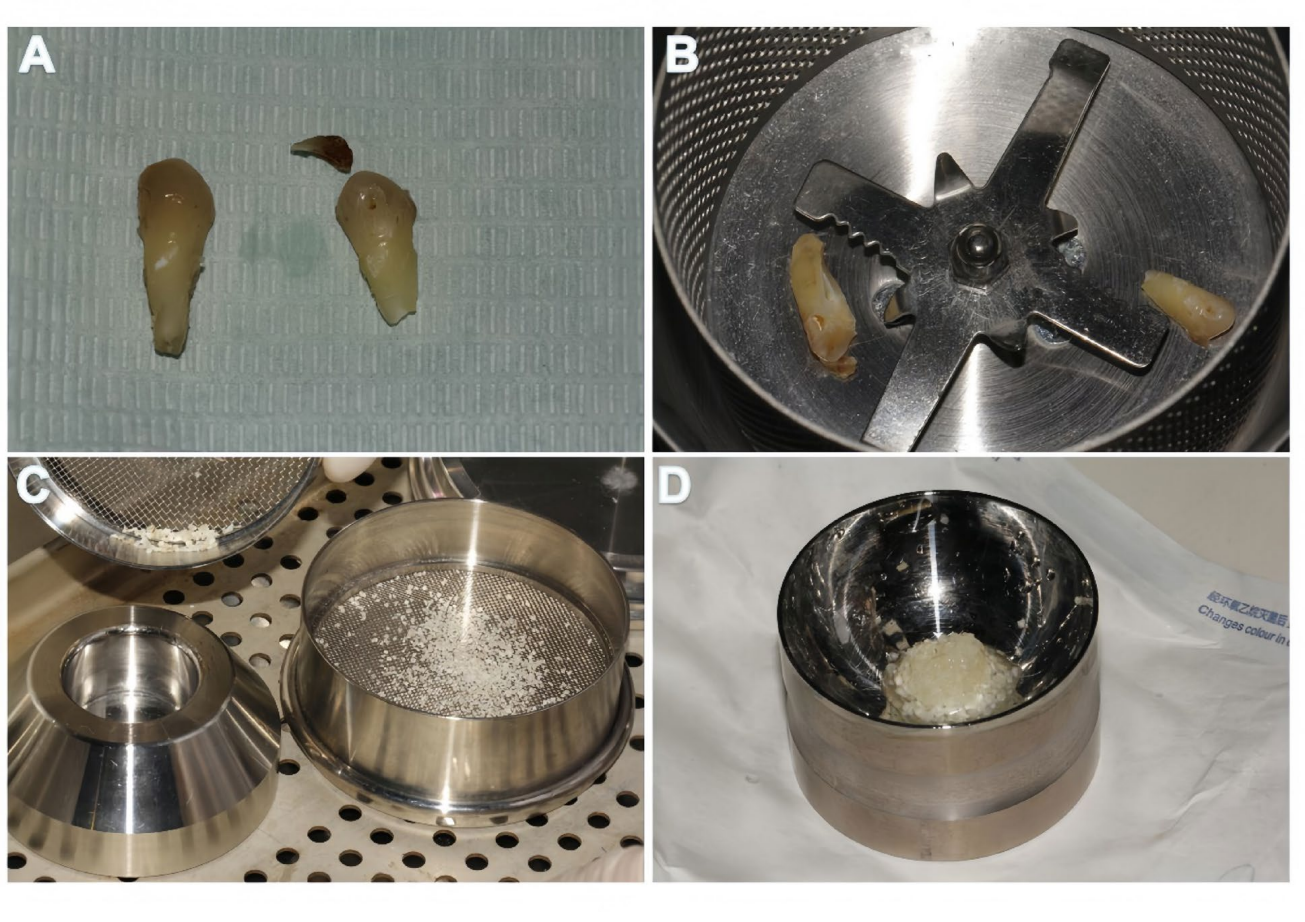
Preparation procedure for the autogenous demineralized dentin matrix (DDM). (**A**) The extracted non-restorable tooth was thoroughly debrided, removing calculus, periodontal ligament, and restorative materials. (**B**) The tooth was processed into a particulate graft using the Chairside DDM device. (**C**) Macroscopic view of the final DDM particles, showing a cortico-cancellous-like structure. (**D**) Detailed view of the DDM graft ready for clinical application. Note: DDM, autogenous demineralized dentin matrix

The extracted tooth was mechanically cleaned to remove calculus, carious tissue, and periodontal ligament remnants. It was then processed using a specialized autogenous tooth bone graft material preparation system (TSBK-ATB-01, Beijing Topstissue Biotechnology Co., Ltd., Beijing, China) to obtain dentin particles (400–800 μm). The particles underwent partial demineralization and sterilization according to the manufacturer’s protocol. Finally, the graft material was rinsed with saline. The prepared DDM was densely packed into the socket (Fig. 1).

#### Control group (DBBM)

**Fig. 2:**
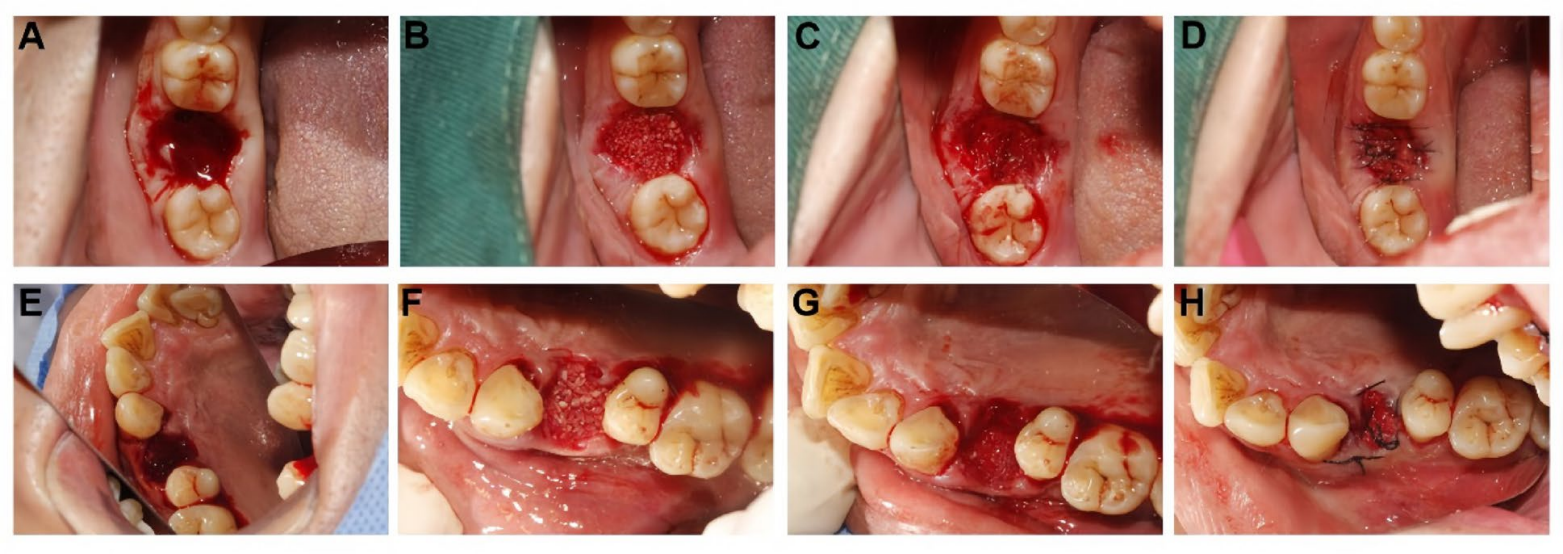
Standardized surgical protocols for ridge preservation in the test and control groups. (**A**–**D**) Test group (DDM): (**A**) Occlusal view of the fresh extraction socket after debridement. (**B**) The socket was densely packed with autogenous demineralized dentin matrix (DDM). (**C**) A collagen membrane was placed to cover the graft material. (**D**) Tension-free primary closure was achieved with interrupted sutures. (**E**–**H**) Control group (DBBM): (**E**) Prepared extraction socket in the control group. (**F**) Grafting with deproteinized bovine bone mineral (DBBM). (**G**) Application of the collagen membrane. (**H**) Final suturing. Note: DDM: autogenous demineralized dentin matrix; DBBM: deproteinized bovine bone mineral

The socket was grafted with deproteinized bovine bone mineral (Bio-Oss®). In both groups, the graft material was covered with a resorbable collagen membrane (Bio-Gide®, Geistlich Pharma AG, Wolhusen, Switzerland) and stabilized with crisscross sutures using non-resorbable thread. The surgical procedure is illustrated in Fig. 2.

### Clinical assessment

Soft tissue healing was evaluated at 7 days, 3 months, and 6 months post-surgery. Healing was assessed using the healing index described by Landry et al. [14]. “The healing index rates surgical sites based on tissue color, bleeding response, presence of granulation tissue, and incision margin closure. The scoring criteria are as follows: Score 1 (Very Poor): Massive inflammation, tissue necrosis, or suppuration. Score 2 (Poor): Significant redness, bleeding on palpation, and granulation tissue present. Score 3 (Good): Mild inflammation, no bleeding on palpation, and partial wound closure. Score 4 (Very Good): Minimal inflammation and complete wound closure. Score 5 (Excellent): No inflammation, healthy pink tissue, and perfect healing”.

This index evaluates tissue color, bleeding response, presence of granulation tissue, and incision margin, scoring the healing quality from 1 (very poor) to 5 (excellent).

### Radiographic evaluation

CBCT scans were obtained for all patients at three time points: immediately after surgery (T0) and 3 months (T1) and 6 months (T2) post-surgery. The DICOM datasets were imported into medical image processing software (Mimics 21.0, Materialise, Leuven, Belgium) for 3D reconstruction and analysis.

#### Image reorientation and standardization

**Fig. 3:**
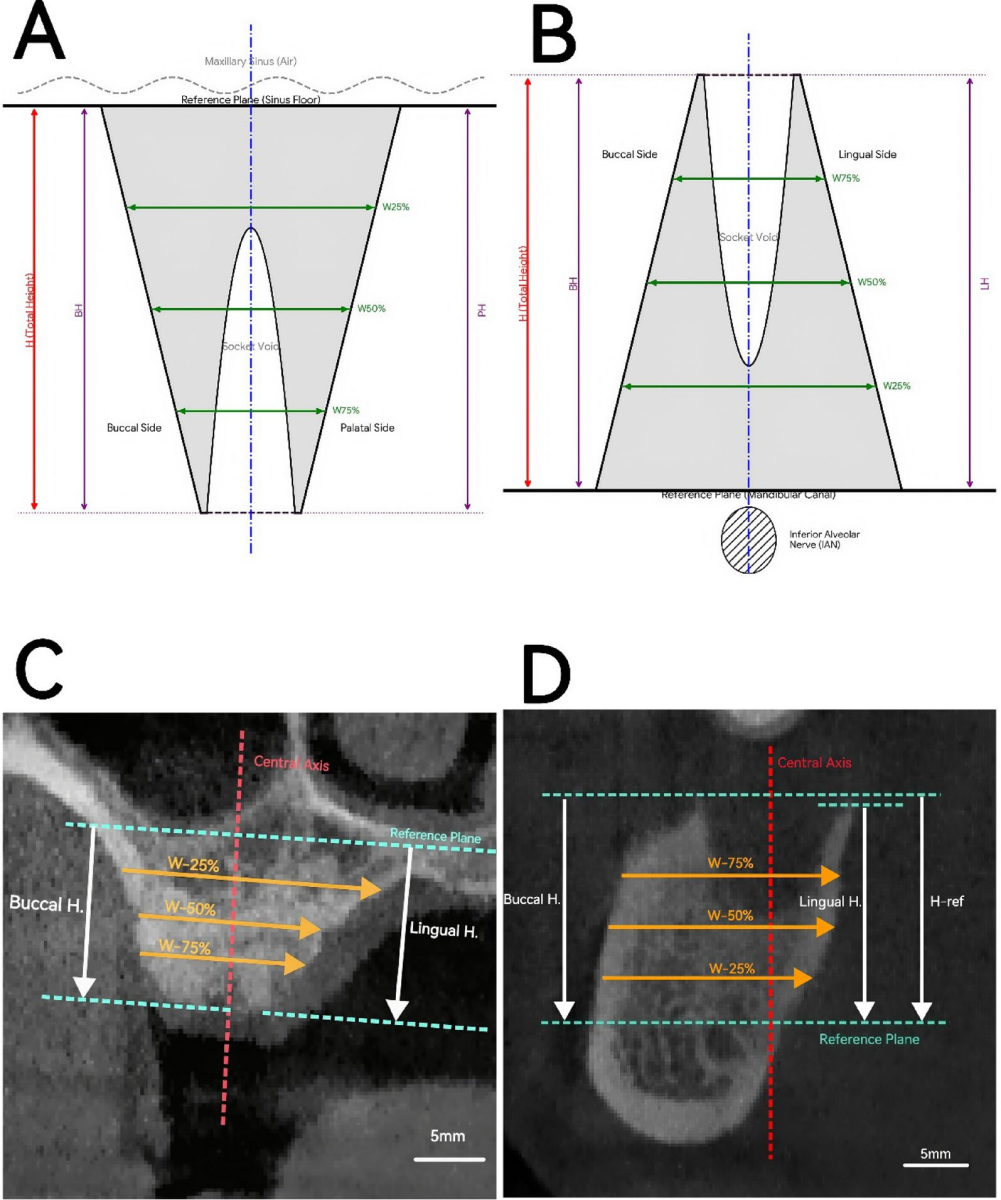
Schematic diagrams and representative CBCT images illustrating the linear measurement methodology. (**A**, **B**) Schematic illustrations defining the standardized anatomical reference planes and measurement landmarks. (**A**) Maxilla: The reference plane (black line) is tangential to the maxillary sinus floor. Palatal height (PH) and buccal height (BH) are measured downwards. (**B**) Mandible: The reference plane is tangential to the superior border of the mandibular canal (containing the IAN). Lingual height (LH) and buccal height (BH) are measured upwards. (**C**, **D**) Representative CBCT cross-sectional images demonstrating the clinical application of the method. (**C**) Maxillary grafted site at 3 months (T1), where the visible high-density graft material maintains the alveolar ridge dimensions. (**D**) Mandibular site at baseline (T0), showing the mandibular canal (arrowhead) as the stable reference. Measurement protocol: In all panels, a basal reference plane (solid black line in schematics; cyan dashed line in CBCTs) and a central axis (vertical dashed line) perpendicular to the plane were constructed. The initial total height (H_ref) was measured from the reference plane to the mid-crestal point at baseline. Alveolar ridge widths (W) (green arrows in schematics; yellow arrows in CBCTs) were measured perpendicular to the central axis at 25% (W_25%), 50% (W_50%), and 75% (W_75%) of the H_ref from the reference plane. Scale bar: 5 mm. Note: IAN: inferior alveolar nerve; PH: palatal height; BH: buccal height; LH: lingual height; H_ref: initial ridge height; W: ridge width; T0: immediately post-surgery; T1: 3 months post-surgery; T2: 6 months post-surgery

To minimize measurement errors caused by variations in head positioning and to ensure longitudinal consistency of the measurement planes, a standardized reorientation procedure was performed based on stable anatomical landmarks, following the protocols described by Chappuis et al. [15] and Timock et al. [16]. Using the “Reslice” function in the multi-planar reconstruction (MPR) view, the coordinate system was adjusted as follows:

- *Horizontal alignment*: The superior border of the mandibular canal (for mandibular sites) or the floor of the maxillary sinus (for maxillary sites) was aligned parallel to the horizontal axis to serve as a stationary reference plane (Fig. 3A, B).
- *Vertical alignment*: The vertical axis was adjusted to pass through the center of the extraction socket and aligned parallel to the long axis of the socket (or the roots of adjacent teeth).
- *Cross-sectional view*: The sagittal axis was adjusted perpendicular to the curvature of the alveolar ridge to obtain a true buccolingual cross-sectional slice (Fig. 3C, D).

#### Measurement protocol

To accommodate anatomical variations in initial bone height [17] and to prevent measurement bias caused by vertical ridge resorption, a proportional measurement protocol was adopted.

At baseline (T0), the total ridge height (H_ref) was measured from the fixed reference plane to the midpoint of the alveolar crest. Based on H_ref, three standardized horizontal levels were defined (Fig. 3A, B):

- *Coronal level*: Located at 75% of H_ref from the reference plane.
- *Middle level*: Located at 50% of H_ref from the reference plane.
- *Apical level*: Located at 25% of H_ref from the reference plane.

This multi-level linear measurement approach aligns with methodologies employed in previous ridge preservation studies [12, 13]. The specific vertical coordinates (in millimeters) calculated at T0 were recorded and strictly applied to the T1 and T2 scans. This ensured that all horizontal width measurements were performed at identical spatial coordinates relative to the fixed reference plane throughout the study period.

#### Outcome variables

The following dimensional parameters were measured on the standardized cross-sectional slices (Fig. 3C, D):

- *Vertical bone height*: Measured as the perpendicular distance from the reference plane to the most coronal point of the buccal bone plate (BH) and the lingual/palatal bone plate (LH or PH).
- *Horizontal bone width*: Measured at the coronal (75%), middle (50%), and apical (25%) levels defined above.

#### Intra-examiner reliability

All measurements were performed by a single calibrated examiner. To assess the intra-examiner reliability, 20% of the samples were randomly selected and re-measured two weeks after the initial measurement. The Intra-class Correlation Coefficient (ICC) was calculated [18]. The results showed an ICC > 0.9, indicating excellent reliability.

### Statistical analysis

All statistical analyses were performed using IBM SPSS Statistics for Windows, Version 27.0 (IBM Corp., Armonk, NY, USA). The normality of the data distribution was assessed using the Shapiro–Wilk test. Continuous variables with a normal distribution are expressed as mean ± standard deviation (SD), and differences between the test and control groups were analyzed using the independent samples *t*-test. Levene’s test was used to assess the homogeneity of variances. For variables that did not follow a normal distribution, data are presented as median (interquartile range [IQR]), and the non-parametric Mann–Whitney U test was employed for group comparisons. Categorical variables (e.g., gender) were compared using the Chi-square test or Fisher’s exact test. To evaluate intra-examiner reliability, the Intra-class Correlation Coefficient (ICC) was calculated based on duplicate measurements of 20% of the samples. A two-tailed *p*-value of < 0.05 was considered statistically significant.

## Results

### Baseline characteristics of the study population

A total of 50 patients were enrolled and randomized into the test group (DDM, *n* = 25) and the control group (DBBM,* n* = 25). The study cohort consisted of 21 males and 29 females, with an age range of 21 to 70 years (mean age: 47.2 ± 13.6 years). There were no statistically significant differences between the two groups regarding age, gender distribution, jaw position, or tooth type at baseline (*p* > 0.05). Detailed demographic and clinical characteristics are presented in Table 1.

**Table 1:** Demographic characteristics of the study population at baseline

Characteristic	Test group (DDM) (*n* = 25)	Control group (DBBM) (*n* = 25)	Total (*n* = 50)	*p*-value
Age (years)				
Range	21–70	26–69	21–70	–
Mean ± SD	46.1 ± 14.2	48.2 ± 13.3	47.2 ± 13.6	0.595 †
Gender, *n* (%)				0.086 ‡
Male	14 (56.0%)	7 (28.0%)	21 (42.0%)	
Female	11 (44.0%)	18 (72.0%)	29 (58.0%)	
Tooth position (Jaw), *n* (%)				> 0.999 ‡
Maxilla	11 (44.0%)	10 (40.0%)	21 (42.0%)	
Mandible	14 (56.0%)	15 (60.0%)	29 (58.0%)	
Tooth type,* n* (%)				> 0.999 ‡
Premolar	4 (16.0%)	5 (20.0%)	9 (18.0%)	
Molar	21 (84.0%)	20 (80.0%)	41 (82.0%)	

Values are presented as *n* (%) for categorical variables and mean ± standard deviation (SD) for continuous variables. Abbreviations: DDM: autogenous demineralized dentin matrix; DBBM: deproteinized bovine bone mineral; SD: standard deviation. Statistical analysis: † *p*-values were calculated using the independent samples *t*-test. ‡ *p*-values were calculated using the Chi-square test or Fisher’s exact test

**Fig. 4:**
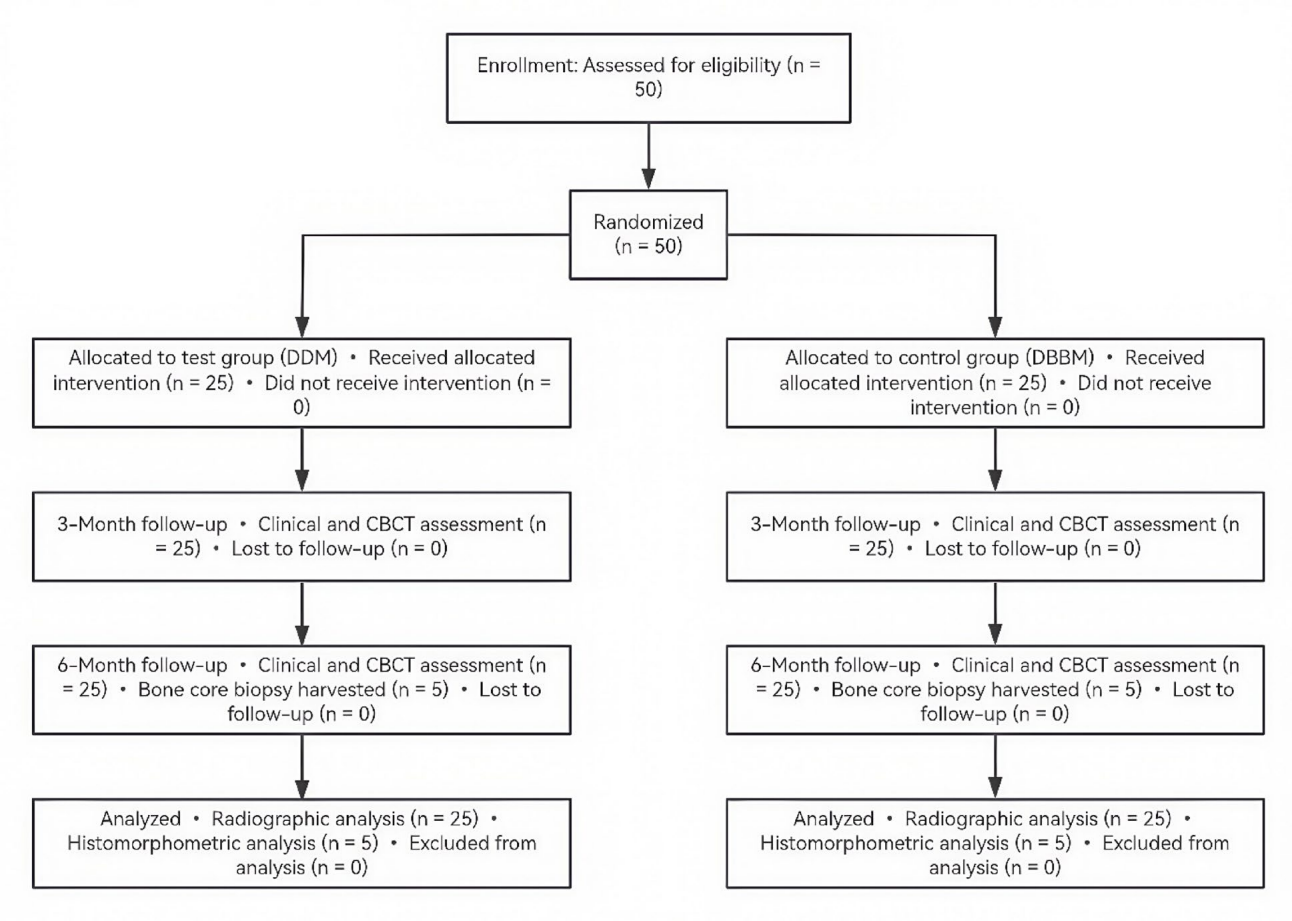
CONSORT flow diagram showing patient enrollment, allocation, follow-up, and analysis. Note: DDM: autogenous demineralized dentin matrix; DBBM: deproteinized bovine bone mineral; n: number of patients

All participants completed the prescribed follow-up protocol at 3 and 6 months post-surgery, with no dropouts recorded. A subset of 10 patients (5 from each group) underwent bone core biopsy for histological analysis. The detailed flow of patient recruitment, allocation, and analysis is illustrated in Fig. 4.

### Dimensional changes at 3 months post-surgery

At 3 months post-surgery, varying degrees of alveolar ridge resorption were observed in both groups.

Regarding horizontal dimensional changes, the Mann–Whitney U test revealed a statistically significant difference in width reduction at the middle level (50% level) (*p* = 0.006). The test group exhibited significantly greater bone loss (0.77 ± 0.59 mm) compared with the control group (0.44 ± 0.45 mm) in this region.

At the apical level (25% level), the independent-samples *t*-test showed a higher width reduction in the test group (0.55 ± 0.43 mm) compared with the control group (0.36 ± 0.26 mm), but the difference was not statistically significant (*p* = 0.069).

No statistically significant differences were observed regarding width reduction at the coronal level (75% level) (test: 1.16 ± 0.84 mm vs. control: 1.26 ± 1.39 mm; *p* = 0.684).

Regarding vertical bone resorption, no significant differences were found between the two groups. The buccal height reduction was similar (test: 1.50 ± 1.19 mm vs. control: 1.06 ± 0.75 mm; *p* = 0.197), as was the lingual height reduction (test: 0.91 ± 0.82 mm vs. control: 0.85 ± 0.67 mm; *p* = 0.846). Details of the dimensional changes are summarized in Table 2.

**Table 2:** Dimensional changes in alveolar ridge height and width at 3 and 6 months post-surgery

Variable	Timepoint	Test group (DDM)	Control group (DBBM)	*p*-value
		(*n* = 25) Mean ± SD	(*n* = 25) Mean ± SD	
Vertical bone resorption (mm)				
Buccal height	3 Months	1.50 ± 1.91	1.06 ± 0.75	0.197
	6 Months	2.12 ± 1.30	1.72 ± 0.84	0.205 †
Lingual height	3 Months	0.91 ± 0.82	0.85 ± 0.67	0.846 †
	6 Months	1.47 ± 0.83	1.21 ± 0.68	0.238
Horizontal width reduction (mm)				
Coronal level (75%)	3 Months	1.16 ± 0.84	1.26 ± 1.39	0.684 ‡
	6 Months	2.10 ± 1.71	1.95 ± 1.59	0.628 ‡
Middle level (50%)	3 Months	0.77 ± 0.59	0.44 ± 0.45	0.006 ‡**
	6 Months	1.42 ± 1.10	1.14 ± 0.74	0.426 ‡
Apical level (25%)	3 Months	0.55 ± 0.43	0.36 ± 0.26	0.069
	6 Months	0.85 ± 0.49	0.73 ± 0.51	0.118 ‡

Data are presented as mean ± standard deviation (SD). All measurements are in millimeters (mm). Positive values indicate bone loss (reduction). Abbreviations: DDM: autogenous demineralized dentin matrix; DBBM: deproteinized bovine bone mineral. Statistical analysis: † *p*-values were calculated using the independent-samples *t*-test. ‡ *p*-values were calculated using the Mann–Whitney U test. * *p* < 0.05 and ** *p* < 0.01 indicate statistical significance

### Dimensional changes at 6 months post-surgery

At the 6-month follow-up, the dimensional changes stabilized, and no statistically significant differences were found between the groups across all measured parameters.

Regarding vertical dimensions, independent-samples *t*-tests indicated comparable outcomes for buccal height reduction (2.12 ± 1.30 mm vs. 1.72 ± 0.84 mm;* p* = 0.205) and lingual height reduction (1.47 ± 0.83 mm vs. 1.21 ± 0.68 mm;* p* = 0.238).

Regarding horizontal dimensions, Mann–Whitney U tests demonstrated no significant differences in width reduction at any level. Specifically, the reduction at the coronal level (75%) was similar between the test and control groups (2.10 ± 1.71 mm vs. 1.95 ± 1.59 mm; *p* = 0.628), as was the reduction at the middle level (50%) (1.42 ± 1.10 mm vs. 1.14 ± 0.74 mm; *p* = 0.426) and the apical level (25%) (0.85 ± 0.49 mm vs. 0.73 ± 0.51 mm; *p* = 0.118). These findings suggest that despite the accelerated initial remodeling observed in the DDM group, the long-term dimensional stability at 6 months was comparable with that of the control group. Detailed results are shown in Table 2.

### Clinical soft tissue healing

Postoperative healing was uneventful in both groups, with no signs of severe infection or graft rejection. At 7 days post-surgery, the test group (DDM) exhibited a slightly higher mean healing score (3.88 ± 0.33) compared with the control group (DBBM) (3.76 ± 0.52), as assessed by the Landry Wound Healing Index (Table 3). The higher scores in the DDM group were characterized by better soft tissue closure and less marginal inflammation, although the difference was not statistically significant (*p* = 0.419).

**Table 3:** Clinical wound healing scores (Landry Index) at 7 days post-surgery

Group	*n*	Healing score (Mean ± SD)	Median (IQR)	*p*-value
Test group (DDM)	25	3.88 ± 0.33	4.0 (4.0–4.0)	0.419
Control group (DBBM)	25	3.76 ± 0.52	4.0 (4.0–4.0)	

Data are presented as mean ± standard deviation (SD) and median (interquartile range, IQR). Abbreviations: SD: standard deviation; IQR: interquartile range; DDM: autogenous demineralized dentin matrix; DBBM: deproteinized bovine bone mineral. Statistical Analysis: *p*-values were calculated using the Mann–Whitney U test

### Clinical observations and implant stability

**Fig. 5:**
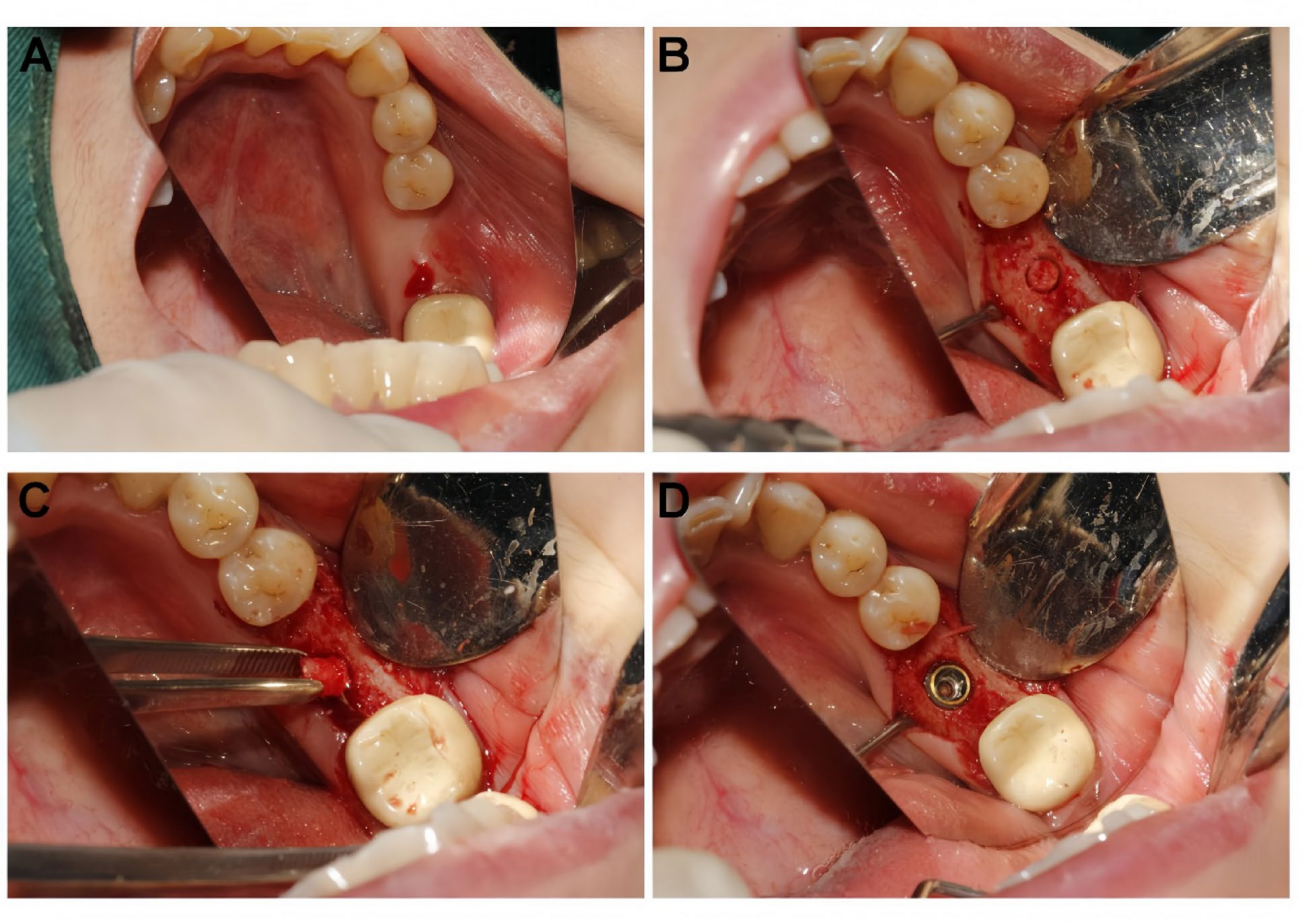
Clinical outcomes and re-entry surgery at 6 months post-surgery. (**A**) Clinical view at 6 months showing uneventful soft tissue healing and preservation of the alveolar ridge contour. (**B**) Flap reflection revealed a wide alveolar ridge with newly regenerated hard tissue suitable for implant placement. (**C**) A bone core biopsy was harvested from the center of the grafted site using a trephine bur for histological analysis (representative case). (**D**) Successful placement of the dental implant with satisfactory primary stability

At 6 months post-surgery, clinical re-entry was performed in patients who returned to our center for implant placement. Upon flap reflection, the extraction sockets in both groups were completely filled with newly regenerated hard tissue. The alveolar ridges appeared well-preserved with sufficient width and height for implant placement (Fig. 5A, B).

Histological bone core biopsies were harvested from 10 representative patients (5 in the DDM group and 5 in the DBBM group) who provided additional consent.

In these treated cases, all implants were successfully placed with satisfactory primary stability. No surgical complications were observed during the procedure (Fig. 5D).

### Histological and immunohistochemical analysis

Histological evaluation of the core biopsies at 6 months revealed distinct healing patterns between the two groups.

In the test group (DDM), HE staining demonstrated that the residual graft particles were undergoing active remodeling. The particles were intimately surrounded by mature new bone (NB) and highly vascularized connective tissue (CT). Immunohistochemical analysis further confirmed the regenerative activity. Ki-67 staining revealed a dense distribution of positive cells within the matrix and vascular endothelium (BV), indicating active tissue turnover and angiogenesis. Additionally, SATB2 staining showed strong nuclear expression in the osteoblasts adjacent to the new bone matrix (NB), confirming robust osteogenic differentiation.

**Fig. 6:**
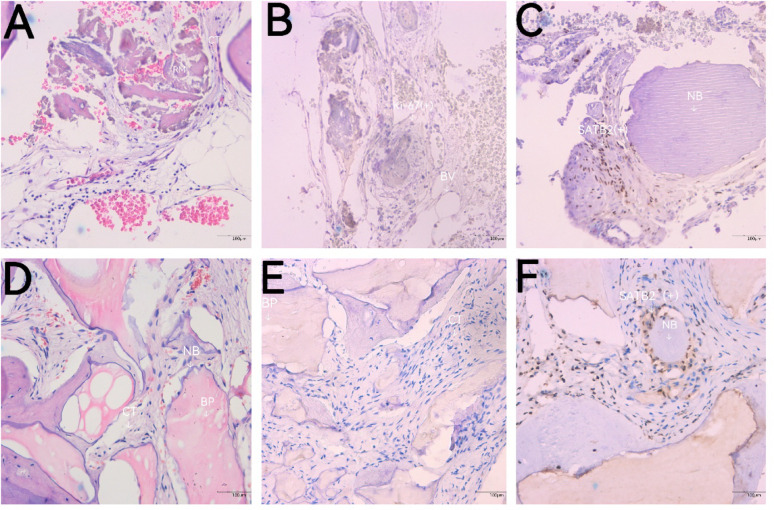
Histological and immunohistochemical evaluation of bone regeneration at 6 months postoperation. (**A**–**C**) Test group (DDM): (**A**) H&E staining showing residual dentin matrix (RM) undergoing remodeling, surrounded by mature new bone (NB) and vascularized connective tissue (CT). (**B**) Ki-67 staining showing positive cells (brown nuclei, arrows) distributed in the stroma and vascular endothelium (BV). (**C**) SATB2 staining exhibiting strong nuclear expression (arrows) in osteoblasts clustered adjacent to the new bone matrix (NB). (**D**–**F**) Control group (DBBM): (**D**) H&E staining showing new bone (NB) formation along the surface of DBBM particles (BP). (E) Ki-67 staining showing a quiescent state with minimal positive expression in the connective tissue (CT). (F) SATB2 staining showing SATB2-positive osteoblasts (arrows) lining the new bone (NB) around the graft particles (BP). Original magnification × 200. Abbreviations: NB: new bone; CT: connective tissue; BV: blood vessel; BP: bone substitute particle (or DBBM particle); RM: residual dentin matrix. Scale bar: 100 μm

In contrast, the control group (DBBM) showed new bone (NB) formation primarily along the surface of the DBBM particles (BP). The tissue appeared to be in a relatively quiescent state, as evidenced by minimal Ki-67 positive expression in the connective tissue (CT). SATB2-positive osteoblasts were observed lining the new bone (NB) surrounding the graft particles (Fig. 6).

## Discussion

This randomized controlled trial aimed to evaluate the clinical, radiographic, and histological efficacy of autogenous demineralized dentin matrix (DDM) compared with deproteinized bovine bone mineral (DBBM) for alveolar ridge preservation (ARP). It is well-established that unassisted socket healing leads to significant dimensional loss, particularly in the horizontal aspect [5, 19]. Our findings demonstrate that, while both DDM and DBBM successfully limited this resorption, they exhibited distinct healing kinetics: DDM showed faster dimensional contraction in the early healing phase (3 months) but achieved alveolar ridge volume maintenance comparable to DBBM at the 6-month endpoint. Histological evidence further confirmed the superior biocompatibility and remodeling potential of DDM, supporting its use as a highly promising autogenous bone substitute.

The disparity in dimensional kinetics was a key observation. Our CBCT analysis revealed that at 3 months post-surgery, the DDM group showed significantly greater horizontal resorption at the mid-socket level (50%) compared with the DBBM group. This finding aligns with previous observations [20, 21], indicating that DDM undergoes active remodeling at an early stage. This early volumetric reduction reflects physiological “creeping substitution” rather than treatment failure. Because DDM is an organic matrix composed primarily of type I collagen, its degradation rate is intimately linked to the rate of new bone formation. In contrast, DBBM, serving as a purely osteoconductive inorganic xenograft, undergoes negligible resorption [3, 22], thus presenting better volumetric stability in the early phase. However, by 6 months, no statistically significant differences in vertical or horizontal dimensions between the two groups were found, suggesting that DDM achieves long-term dimensional stability following its initial remodeling phase.

Histological evaluation further elucidated the distinct bone healing patterns between the two groups. Our histological sections revealed that DBBM particles were primarily surrounded by new bone trabeculae or bridged by connective tissue, with no evident signs of particle resorption, confirming their role as a stable osteoconductive scaffold. In contrast, the DDM group exhibited a dynamic remodeling process: DDM particles were intimately fused with new bone, and osteoclastic lacunae were visible on the surface of some particles, indicating that the material was undergoing biodegradation and gradual conversion into host bone. This finding aligns with the recent histomorphometric analysis by Beca-Campoy et al. [21], who reported that autogenous tooth grafts promote high-quality de novo mineralized bone formation. Inchingolo et al. [23] attributed this superior tissue response to the type I collagen scaffold and endogenous growth factors (e.g., BMP-2) retained within the dentin matrix [23–25]. These bioactive molecules not only recruit osteoblasts but may also accelerate the transition from woven to lamellar bone, thereby providing a more vitalized bone bed for implants rather than a mere inorganic mineral filler.

Regarding radiographic appearance and biological mechanisms, DDM demonstrated distinct advantages. In the 6-month CBCT images, DBBM particles presented as clear, high-density radiopaque structures with distinct boundaries from the surrounding bone. Conversely, the radiodensity of the DDM graft gradually merged with that of the host bone, resulting in blurred boundaries. This is not an artifact but direct evidence of the “tissue integration” phenomenon described by Minetti et al. [26]. This radiographic “blurring” reflects the active participation of DDM in bone remodeling. Previous studies have highlighted that dentin matrix, processed through specific demineralization, retains endogenous growth factors (such as BMP-2 and TGF-β), endowing the material with osteoinductive potential [23, 26]. This explains our histological findings of active new bone deposition and collagen fiber ingrowth directly onto DDM surfaces, contrasting with the primary scaffolding role of DBBM.

Furthermore, the standardized radiographic measurement protocol employed in this study enhanced data reliability. Previous studies often measured width solely at the alveolar crest, which is susceptible to bias from vertical resorption. To overcome this limitation, we established a coordinate system based on fixed anatomical landmarks (mandibular canal/maxillary sinus floor), referencing protocols by Timock et al. [16] and Chappuis et al. [15]. By implementing an “initial height percentage protocol (H_ref-based protocol)”, measurements at T0, T1, and T2 were consistently performed at the same anatomical levels (25%, 50%, and 75%). This approach eliminated measurement-level shifts caused by crestal resorption, thereby more accurately quantifying the three-dimensional remodeling patterns at different depths of the socket.

It is noteworthy that in some cases, vertical resorption exceeding 2 mm was observed in both groups. This finding may be attributed to the predominance of thin buccal bone phenotypes in our sample. As demonstrated by Araújo et al. [3], the resorption of the bundle bone is a physiological inevitability following extraction, regardless of the grafting material used. Furthermore, strict CBCT measurements based on a fixed reference plane may capture minute crestal dimensional changes more sensitively than direct clinical measurements used in earlier studies. Despite this vertical reduction, the preserved ridge width remained sufficient for implant placement in all cases, confirming the clinical utility of the procedure.

Clinical significance and strategic value: Beyond its biological advantages, DDM demonstrates significant clinical utility. As an autogenous material, DDM exhibits excellent biocompatibility, completely eliminating the potential risks of disease transmission and immune rejection associated with xenografts, a safety profile confirmed by prospective randomized controlled trials [27]. Regarding subsequent implant therapy, Kim [28] reported that chairside-prepared autogenous tooth grafts induce favorable new bone formation and provide rigid bone support, ensuring high long-term survival rates of implants under functional loading. Furthermore, a recent evaluation of human tooth properties by Desarda et al. [29] further validated its physicochemical superiority as a grafting material. This waste-to-resource strategy not only reduces medical waste through the recycling of autogenous tissue but also presents a scientifically sound, efficient, and cost-effective solution for alveolar ridge preservation.

The limitations of this study include the relatively small sample size and the limited follow-up period of 6 months. While DDM demonstrated promising osteogenic potential, its long-term marginal bone stability under implant loading requires further clinical tracking. In conclusion, DDM offers a predictable, biologically superior, and cost-effective solution for alveolar ridge preservation.

## Conclusions

Within the limitations of this study, the findings indicate that autogenous demineralized dentin matrix (DDM) achieves alveolar ridge dimensional preservation comparable to deproteinized bovine bone mineral (DBBM), despite exhibiting more active remodeling kinetics in the early healing phase. Histological and radiographic evidence confirms that DDM possesses superior osteoinductive potential and tissue integration capabilities, promoting high-quality de novo bone formation. Considering its safety profile, biological advantages, and cost-effectiveness through the recycling of extracted teeth, DDM represents a viable and sustainable alternative material for clinical alveolar ridge preservation.

## Data Availability

The datasets used and/or analysed during the current study are available from the corresponding author on reasonable request.

## Abbreviations

**ARP**: Alveolar ridge preservation
**BIC**: Bone-implant contact
**BMPs**: Bone morphogenetic proteins
**CBCT**: Cone-beam computed tomography
**DBBM**: Deproteinized bovine bone mineral
**DDM**: Autogenous demineralized dentin matrix
**DICOM**: Digital imaging and communications in medicine
**HA**: Hydroxyapatite
**HE**: Hematoxylin and eosin
**IAN**: Inferior alveolar nerve
**ICC**: Intra-class correlation coefficient
**IQR**: Interquartile range
**MPR**: Multi-planar reconstruction
**OPN**: Osteopontin
**RCT**: Randomized controlled trial
**SD**: Standard deviation
**TCP**: Tricalcium phosphate
**TGF-β**: Transforming growth factor-β
**WHO**: World Health Organization
